# Osteonecrosis of the femoral head due to brucellosis: a case report

**DOI:** 10.1186/s12879-020-4945-8

**Published:** 2020-03-06

**Authors:** Peixu Wang, Wei Sun, Lijun Shi, Tengqi Li

**Affiliations:** 1 Department of Orthopedics, China-Japan Friendship Hospital, China-Japan Friendship Institute of Clinical Medicine, Chinese Academy of Medical Sciences, Peking Union Medical College, Graduate School of Peking Union Medical College, Beijing, 100029 China; 2 Department of Orthopedics, China-Japan Friendship Hospital, National Health and Family Planning Commission of the People’s Republic of China, Beijing, 100029 China; 3 Peking University China-Japan Friendship School of Clinical Medicine, 2 Yinghuadong Road, Chaoyang District, Beijing, 100029 China

**Keywords:** Brucellosis, Osteonecrosis of the femoral head, ONFH

## Abstract

**Background:**

Brucellosis is a zoonotic infection transmitted from infected animals to humans, osteonecrosis of the femoral head (ONFH) is a devastating disease that affects patients’ life with pain, dysfunction of walking and always lead to total hip arthroplasty (THA). We presented a case of ONFH which was very likely due to the infection of Brucella spp.

**Case presentation:**

The patient was a 49 years-old male who was a herder living in Inner Mongolia, the northern part of China. He first showed recurrent fever then presented bilateral hip pain, which was confirmed to be brucellosis and ONFH on the right side of the hip. He was admitted to our center showed bilateral ONFH with the restrictive movement of both hips. We performed THA after it was confirmed that the infection has been cured. The patient can walk with the help of the walker the second day after surgery.

**Conclusion:**

Brucellosis is still a common epidemic disease worldwide, which can lead to many complications, brucellosis arthritis is the most common complication of Brucellosis. Osteonecrosis of the femoral head can also present in the patients with brucellosis. All the patients presented with recurrent fever and hip pain, who is from the epidemic region, should be taken both septic arthritis and ONFH into consideration.

## Background

Brucellosis, caused by Brucella spp., is a zoonotic infection transmitted from infected animals to humans [1]. The most common symptoms of Brucellosis were malaise, night sweats, fever and arthralgias [2]. In China, the number of reported cases of Brucellosis was 513,034 from 1955 to 2014, (3504 every year on average). Most patients were male and the majority of them were farmers or worked in livestock husbandry. Of all the reported cases of Brucellosis, 99.3% were from the provinces of the northern part of China [3].

Osteonecrosis of the femoral head (ONFH) is a devastating disease that affects patients’ life with pain, dysfunction of walking and always lead to total hip arthroplasty (THA) [4]. There is no consensus on the exact etiology of the ONFH, but the most common risk factors of ONFH have been identified: trauma, corticosteroid, and alcoholism [5].

Previous reports focused on brucellosis arthritis secondary to the brucellosis. We presented a case with osteonecrosis of the femoral head that was very likely due to the infection of Brucella spp.

## Case presentation

**Fig. 1:**
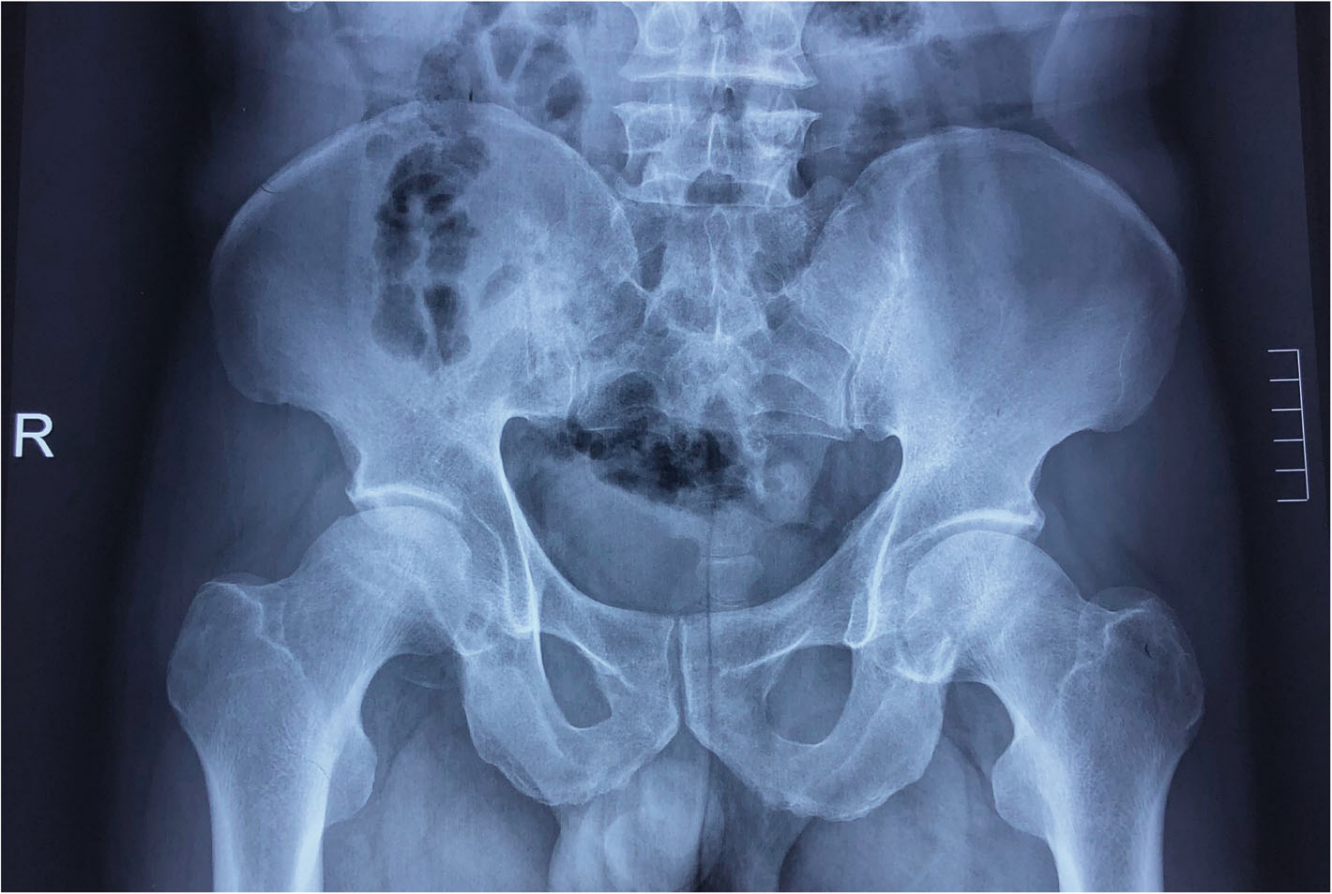
Plain radiograph showed osteonecrosis of the femoral head on the right side of the hip (ARCO Stage III) whereas the left side of the femoral head remains normal (ARCO Stage II)

**Fig. 2:**
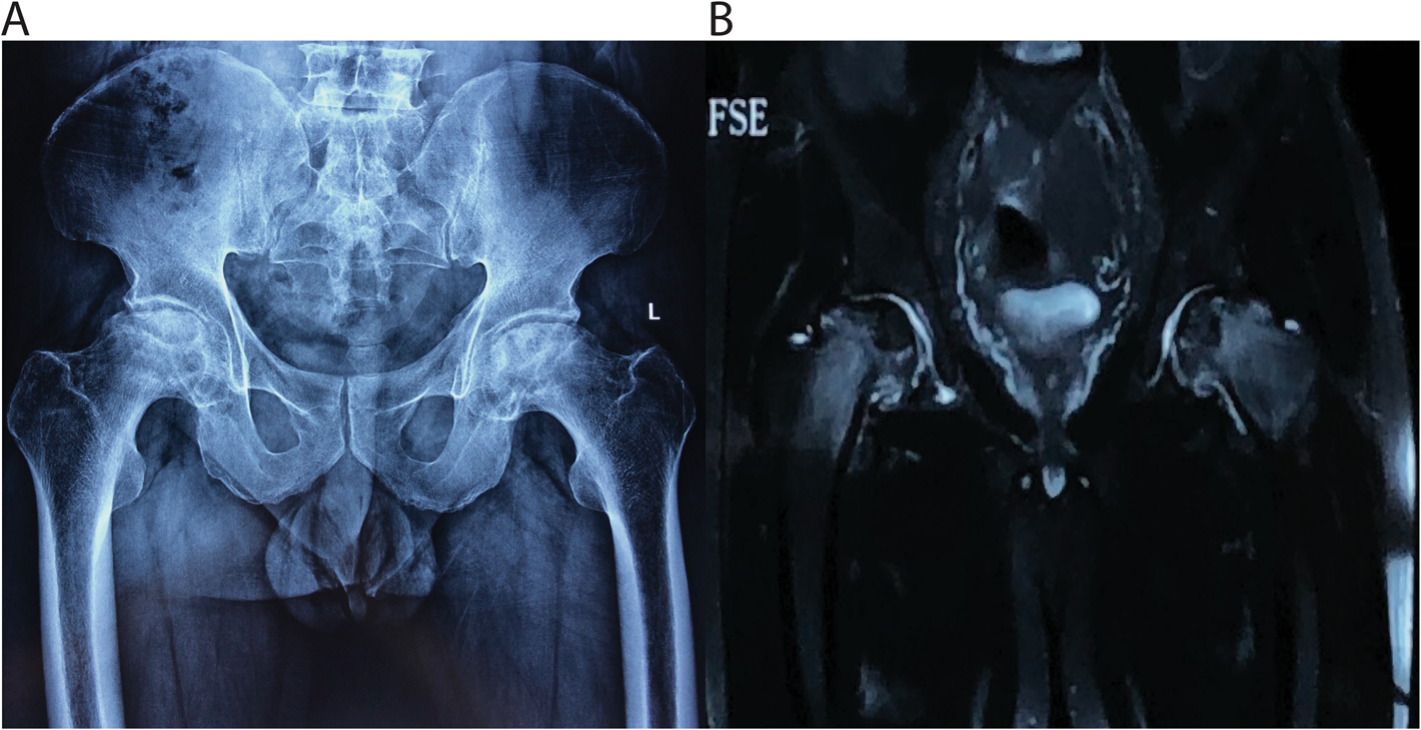
Both plain radiograph (**a**) and MRI (**b**) showed osteonecrosis on the bilateral side of the femoral head 1 year after the first diagnosis

**Fig. 3:**
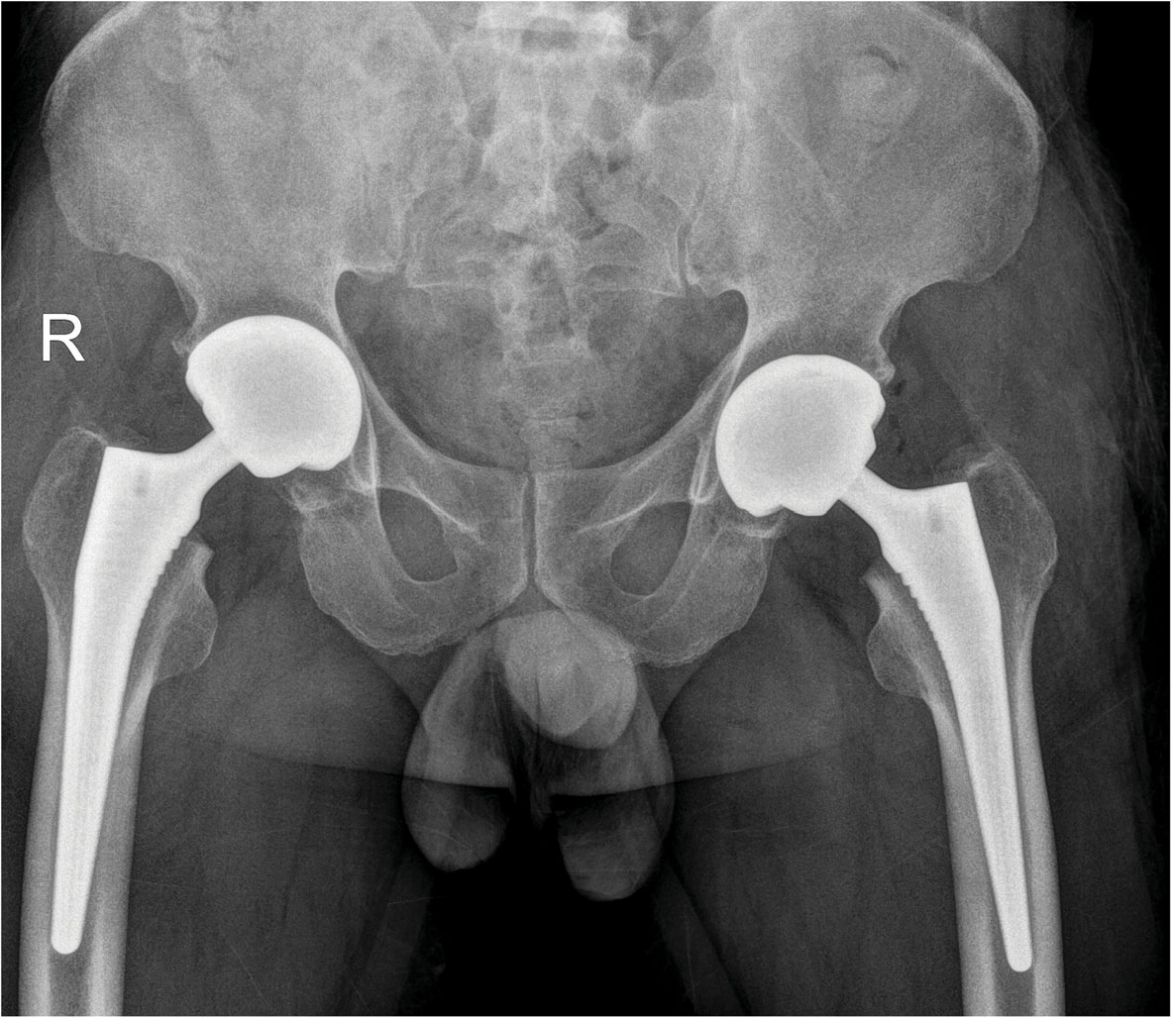
Plain radiograph after THA

The patient was a 49 years-old male who was a herder living in Inner Mongolia, the northern part of China. He did not receive any treatment involving corticosteroid nor did he intake large amounts of alcohol. The patient first presented with recurrent fever. A few days later pain in bilateral sides of hips showed. He was admitted to the local hospital, the experimental test indicated that the patient is infected with Brucella spp., plain radiography showed normal contour of the femoral head on the left side whereas the right side had already developed into ARCO Stage IIIA (Fig. 1), he did not receive standard treatment for Brucellosis at that time. The patient was also diagnosed with spondylitis and have a history of chronic hepatitis B. His symptom of hips aggravated only after a few months and the patient has been treated with doxycycline and rifampin for 6 weeks at the local hospital. Before discharging the Rose-Bengal Plate Agglutination Test (RBPT) showed weakly positive, the Wright test showed a titer of 1:50 and the Cysteine test showed a titer of 1:20. At the time the patient admitted to our center, both sides of the femoral head were aggravated to ARCO Stage IIIC in plain radiography (Fig. 2a), and the necrotic lesion showed in magnetic resonance imaging (MRI) (Fig. 2b). The patient showed limited active and passive hip movement, the abduction and adduction were significantly restricted, Patrick test and Thomas sign were both positive on both sides, Harris Hip Score were 54 on the right side and 45 on the left side. The Agglutination test of Brucella showed weakly positive, and erythrocyte sedimentation rate (ESR), C-reactive protein (CRP) levels and aspartate aminotransferase (AST) were within the normal range. We performed bilateral total hip arthroplasty (THA), during the procedure, we used iodophors soaking the wound for 3 min and then irrigated before and after intramedullary reaming to prevent infection. The procedure was successfully performed (Fig. 3), and we added doxycycline and rifampin to the post-operative period to prevent the relapse of Brucellosis. The patient was able to walk with the help of the walker on the second day of the surgery. The movement of both hips was improved. Harris Hip Score was 80 on the right side and 85 on the left side. All experimental results showed no recurrence of infection after the surgery.

## Discussion and conclusions

The basic mechanism of ONFH is an interruption of circulation to a specific area of the femoral head that eventually aggravated to necrotic [4], but the exact origin remains unknown. The most common risk factors were trauma, corticosteroid, alcoholism [5]. The existing pathogenic mechanisms of bone cell death includes ischemia, direct cellular toxicity, and altered differentiation of mesenchymal stem cells [6].

Brucellosis is still a common epidemic disease worldwide [7]. In developing countries, the disease still has not been controlled effectively [8], and the number of incidence has been rising in China these years [3]. Brucellosis arthritis is the most common complication of Brucellosis, it occurs in up to 70% of patients with brucellosis [9]. The most common site of peripheral arthritis were the knees, hips, and ankles [10].

There were a few articles reporting arthritis caused by Brucellosis, but there is still no report on Brucellosis-induced osteonecrosis. The patient presented with the typical symptom of brucellosis and the experimental test confirmed the infection. The patient also presented with bilateral hip pain, which confirmed to be osteonecrosis of the femoral head. Due to the lateness of the first visit to the hospital, we cannot confirm whether the osteonecrosis was absolutely caused by the infection of Brucella spp. But since the patient has no history of corticosteroid intake nor did he have alcohol abuse, and there was no symptom related to the hip before the infection, we presume the osteonecrosis of the femoral head was very likely related to the brucellosis on this patient. Whether the osteonecrosis was directly emerged after the infection or converted from septic arthritis also remained unknown.

Previous reports focused on the brucellosis arthritis, which can leave malfunction of the affected joints. After standard six-week treatment, arthritis can be cured, and the movement of the joint can partly or fully recover. But the case we reported should raise the awareness of the ONFH as a complication of the brucellosis, which most likely cannot recover to normal, and eventually lead to total hip arthroplasty.

Therefore the diagnosis of brucellosis is important. We recommend the patient presented with hip pain, who is from the epidemic region, should be taken both septic arthritis and ONFH into consideration. As for the diagnosis of brucellosis arthritis, it should be diagnosed using blood culture and polymerase chain reaction (PCR) analysis using peripheral blood or synovial fluid. Bone marrow culture can also be conducted in patients with negative serology. With regard to ONFH, MRI should be conducted at the early stage of the infection. And if the osteonecrosis is presented, conservative treatments or hip persevering surgeries should be the first choice of treatment.

## Data Availability

All data and materials are available with the first author.

## Abbreviations

AST: Aspartate aminotransferase (AST)
CRP: C-reactive protein
ESR: Erythrocyte sedimentation rate
MRI: Magnetic resonance imaging
ONFH: Osteonecrosis of the femoral head
PCR: Polymerase chain reaction
RBPT: Rose-Bengal Plate Agglutination Test
THA: Total hip arthroplasty
